# When water interacts with temperature: Ecological and evolutionary implications of thermo‐hydroregulation in terrestrial ectotherms

**DOI:** 10.1002/ece3.5440

**Published:** 2019-08-02

**Authors:** David Rozen‐Rechels, Andréaz Dupoué, Olivier Lourdais, Simon Chamaillé‐Jammes, Sandrine Meylan, Jean Clobert, Jean‐François Le Galliard

**Affiliations:** ^1^ Sorbonne Université, UPEC, CNRS, IRD INRA Institut d'Écologie et des Sciences de l'Environnement, IEES Paris France; ^2^ UMR 5321 CNRS-Université Toulouse III Paul Sabatier Station d'Écologie Théorique et Expérimentale Moulis France; ^3^ UMR 7372 CNRS-ULR Centre d'Études Biologiques de Chizé Villiers en Bois France; ^4^ School of Life Sciences Arizona State University Tempe AZ USA; ^5^ CNRS, Univ Montpellier, EPHE, IRD, Univ Paul Valéry Montpellier 3 Centre d'Écologie Fonctionnelle et Évolutive Montpellier France; ^6^ Sorbonne Université ESPE de Paris Paris France; ^7^ École normale supérieure, CNRS, UMS 3194 Centre de recherche en écologie expérimentale et prédictive (CEREEP‐Ecotron IleDeFrance), Département de biologie PSL Research University Saint‐Pierre‐lès‐Nemours France

**Keywords:** behavioral decisions, body temperature, performance curves, physiological adjustments, water balance

## Abstract

The regulation of body temperature (thermoregulation) and of water balance (defined here as hydroregulation) are key processes underlying ecological and evolutionary responses to climate fluctuations in wild animal populations. In terrestrial (or semiterrestrial) ectotherms, thermoregulation and hydroregulation closely interact and combined temperature and water constraints should directly influence individual performances. Although comparative physiologists traditionally investigate jointly water and temperature regulation, the ecological and evolutionary implications of these coupled processes have so far mostly been studied independently. Here, we revisit the concept of thermo‐hydroregulation to address the functional integration of body temperature and water balance regulation in terrestrial ectotherms. We demonstrate how thermo‐hydroregulation provides a framework to investigate functional adaptations to joint environmental variation in temperature and water availability, and potential physiological and/or behavioral conflicts between thermoregulation and hydroregulation. We extend the classical cost–benefit model of thermoregulation in ectotherms to highlight the adaptive evolution of optimal thermo‐hydroregulation strategies. Critical gaps in the parameterization of this conceptual optimality model and guidelines for future empirical research are discussed. We show that studies of thermo‐hydroregulation refine our mechanistic understanding of physiological and behavioral plasticity, and of the fundamental niche of the species. This is illustrated with relevant and recent examples of space use and dispersal, resource‐based trade‐offs, and life‐history tactics in insects, amphibians, and nonavian reptiles.

## 1 INTERPLAY BETWEEN TEMPERATURE AND WATER CONSTRAINTS: WHY SHOULD WE CARE?

Water availability and climate conditions, especially environmental temperature, vary widely in space and time, influence the energy and water budgets of terrestrial and semiterrestrial animals, and drive the evolution of functional adaptations to cope with temperature and water constraints (Angilletta, 2009; McKinley, Martelli, Pennington, Trevaks, & McAllen, 2018; Mole, Rodrigues DÁraujo, van Aarde, Mitchell, & Fuller, 2016; Sears et al., 2016). Comparative physiologists are well aware of the joint effects of physical environmental conditions (e.g., air temperature, atmospheric moisture, radiation) and environmental resources (e.g., free‐standing water, food) on the energy and water budgets of animals (Bradshaw, 2003; Chown & Nicolson, 2004). At the same time, ecologists emphasize that environmental constraints on water, temperature, and energy budget may vary nonindependently in time and space. Ecological studies often reveal spatial covariations in, for example, air temperature, atmospheric moisture, and soil humidity that result from changes in landscape structure and microhabitats, such as the degree of shading or the vegetation type (e.g., in reptiles: Guillon, Guiller, DeNardo, & Lourdais, 2013; Kearney, Shine, & Porter, 2009; Sears et al., 2016). In addition, temporal covariation in air temperature and atmospheric moisture also occurs in the form of correlated daily or seasonal fluctuations in climate conditions (Owen‐Smith & Goodall, 2014), and joint trends in temperature and water availability are also likely across multiple years as a consequence of global climate change (Kelley, Mohtadi, Cane, Seager, & Kushnir, 2015). The human‐induced climate change and increased incidence of extreme warm spells will affect the energy budget and functional performances of most animal species (reviewed in Buckley & Huey, 2016; Sinervo et al., 2010). Concurrent changes in rainfall, water availability, and ambient humidity might accentuate or buffer the ecological consequences of climate warming depending on patterns of change in hydration state at the organism level (Cahill et al., 2012). This emphasizes the need to carefully investigate and account for dual changes in temperature and water availability in the environment.

Water and temperature are also jointly critical for life from the cellular level to whole‐organism performances (Franks, Mathias, & Hatley, 1990). Body temperature influences the speed of enzymatic reactions and the structure of cellular membranes (Angilletta, 2009), and water is the solvent of biochemical reactions and a fluid for nutritional provisioning of cells (Chaplin, 2006). In tandem, temperature and hydration conditions are therefore crucial for biochemical reactions and cell metabolism. Appropriate regulation of body temperature and water balance (movement of water out and into the organism) is therefore critical for organism performance and involves the modulation of a wide range of physiological and behavioral mechanisms through multiple hormone secretions (Bradshaw, 2007). Recent studies have indeed emphasized that the vulnerability of animals to climate change may be the consequence of abnormally high costs resulting from the regulation of their body temperature (thermoregulation, see Box 1) and of their water balance (hydroregulation, see Box 2 and Cahill et al., 2012).

Box 1 **Thermoregulation**

**Figure 1:**
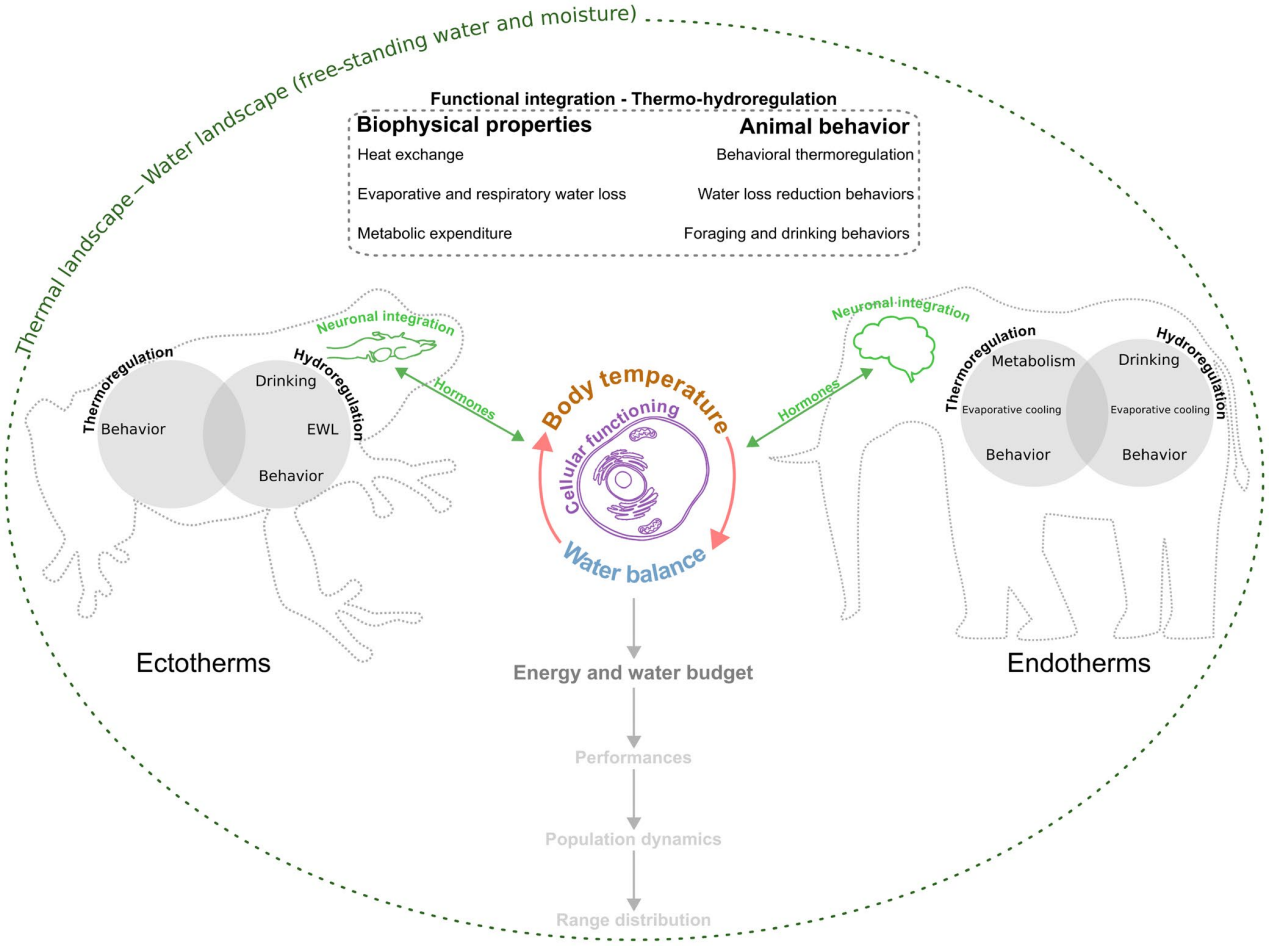
**Heat and water exchange rules in ectotherms and endotherms**. Body temperature and water balance are jointly influenced by heat and water exchanges within the organism and between the organism and its environment. These exchanges are modulated by (i) the biophysical and physiological properties of the organism and by (ii) behavioral strategies. Biophysical properties include morphology, surface properties (skin ultrastructure, fur, feathers, etc.), and metabolic modes. For instance, skin color, thickness, and ultrastructure in reptiles and amphibians determine heating capacity and resistance to water loss. Heat and water exchanges can influence each other; for example, evaporative water loss (EWL) induces heat loss (evaporative cooling) whereas metabolic reactions are a source of heat and water, for example, through fat catabolism. Behavioral components of thermo‐hydroregulation include activity, microhabitat selection, postural adjustments, and drinking and foraging behaviors. Overall, thermo‐hydroregulation strategies differ between ectotherms and endotherms. Ectotherms rely predominantly on behavioral thermoregulation and secondarily on evaporative cooling, most often in wet‐skinned amphibians and during high flight activity in insects, to regulate their body temperature (Stevenson, 1985). Endotherms generate heat and water through metabolism and use evaporative water losses (panting and/or sweating) to cool down their body. The regulation of body temperature and water balance involves neuronal integration and feedback reactions from the central nervous system that orchestrate hormone secretions involved in the maintenance of temperature and water balance homeostasis. Body temperature and water balance regulation determine on the long run the energy and water budget of the individual and ultimately whole‐organism performances (e.g., locomotion, growth, and reproduction), population dynamics, and eventually range distributions

**Figure 2:**
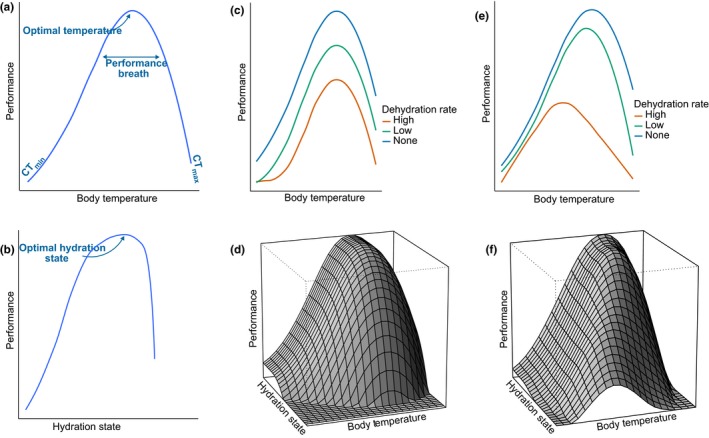
**Performance curves**. A performance curve is an empirical curve describing the relationship between an organism's performances (a functional trait measuring whole‐organism capacity, behavioral trait, or fitness measurement) and the individual state such as its body temperature (thermal performance curve) or its hydration state (hydric performance curve). (a) Hypothetical thermal performance curve. Thermal performance curves are typically characterized by a linear to geometric growth with increasing body temperature until a maximum is reached at an optimal range of temperatures. This is then followed by a rapid performance decrease at body temperatures above the optimum, meaning that a small increase above the optimum can be lethal (Dowd et al., 2015; Huey & Kingsolver, 1989). Critical thermal limits (CTs) are the lower (CT_min_) and upper temperature thresholds (CT_max_) beyond which animals die. (b) Hypothetical hydric performance curve. The hydric performance curves can be calculated from studies of dehydration and describe the relationship between an animal performance and hydration state. Examples of such curves can be found in studies of maximal locomotor capacities in dehydrated amphibians (Anderson & Andrade, 2017; Mitchell & Bergmann, 2016; Preest & Pough, 1989) and of muscular and cognitive performances in endotherms (Cheuvront & Kenefick, 2014). The common pattern is that performances are maximized when water is provided *at libitum* (i.e., optimal hydration state) and decrease rapidly with dehydration state. Hyperhydration can also lead to performance loss due to the mass effects and changes in the osmotic balance, especially in some insects with water excess due to a diet rich in water and high metabolic water production (Chown & Nicolson, 2004). (c–f) Hypothetical thermo‐hydroregulation performance curves. (c, d) Here, performances curves are determined additively by hydration state and body temperature. The optimal body temperature for performance remains the same whatever the hydration state. Performance decreases when temperature and hydration state departs from their optimal values. (e, f) Here, performances curves are determined nonadditively by water balance and body temperature. Optimal body temperature for performance decreases when animals are more dehydrated because dehydration changes the thermal sensitivity of cell and tissue metabolism or the protection against thermal stress (Akerman, Tipton, Minson, & Cotter, 2016)

1 Thermoregulation includes a range of mechanisms involved in the regulation of the body temperature and energy metabolism in endotherms and ectotherms (Figure 1). Body temperature has immediate effects on physiological performances, such as maximal locomotor capacities or energy assimilation, and on survival due to the existence of critical thermal limits. This thermal dependence is illustrated by the concept of the thermal performance curve where a temperature breadth (range where performances >80% of the maximum) is optimal for fitness (Figure 2a). This range must be reached by physiological or behavioral thermoregulation, that is, the costly investment of time and energy into heat production and/or heat exchanges with the environment. To do so, organisms, especially ectothermic animals, rely on behavioral changes in activity patterns, movements, or microhabitat choices (Caillon, Suppo, Casas, Arthur Woods, & Pincebourde, 2014; Sears et al., 2016), behavioral changes in posture or orientation (Barton, Porter, & Kearney, 2014), or anatomical and color changes in the body surface (Stuart‐Fox, Newton, & Clusella‐Trullas, 2017). Endothermic organisms also rely on heat production through metabolism and heat loss through evaporative cooling (Clarke & Rothery, 2008). The thermal quality of the environment is best measured by operative temperatures, steady‐state temperatures of an organism in a particular environment in the absence of evaporative cooling and metabolic heat production, which are determined by local microclimate as well as the physical properties of the organism (Bakken, 1992; Dzialowski, 2005). Thermal constraints are universal and can exist in aquatic and terrestrial environments, but the cost of thermoregulation depends very much on mean and variance of operative temperatures (Sears & Angilletta, 2015; Sears, Raskin, & Angilletta, 2011). Spatial variation in operative temperatures depends on topography and habitat complexity, and temporal variation is often strong but predictable because of daily and seasonal cycles (Paaijmans et al., 2013). Since environmental temperature is a non‐depreciable resource, the evolution of optimal thermoregulation can be described by a cost–benefit model that accounts for the time and energy constraints on thermoregulation effort and heat production (Blouin‐Demers & Nadeau, 2005; Huey & Slatkin, 1976), as well as nonenergetic costs induced by predation or interference competition (Angilletta, 2009).

Box 2 **Hydroregulation**

1 Hydroregulation, defined as the set of behavioral and physiological mechanisms to control water balance and remain hydrated, is one component of osmoregulation, that is, the regulation of ionic concentration such as salts and minerals in body fluids in which water is the solvent. Water balance determines the hydration state of the organism, defined as the volumetric quantity of water (or percentage of body water). There are numerous markers of water balance depending on the model species such as direct measures of water content, or indirect measures, for example, measures of body mass changes or plasma osmolality. Hydroregulation only applies to semiterrestrial organisms living at the transition zone between aquatic and terrestrial environments, like amphibians, and terrestrial animals. The relation between hydration state and whole‐organism performance is complex since fitness effects arise from a deviation from optimal hydration state reflecting cumulative losses and gains of water over several days or weeks. The shape of the whole‐organism hydric performance curves has been little examined relative to thermal performance curves, but there is a strong indication from most published studies for an optimal hydration state that is a critical homeostatic target (Figure 2b). To reach this optimal water balance, hydroregulation involves three major mechanisms: (a) *water conservation processes* such as physiological changes in skin resistance and panting (Tattersall, Cadena, & Skinner, 2006; Wegener, Gartner, & Losos, 2014), behavioral changes in activity and posture (Chown, Sørensen, & Terblanche, 2011; Pintor, Schwarzkopf, & Krockenberger, 2016; Pough, Taigen, Stewart, & Brussard, 1983), and regulation of urine and feces production (Cain, Krausman, Rosenstock, & Turner, 2006) (b) mechanisms to regulate *water intake* through habitat selection and drinking behavior (i.e., free‐standing water intake, Davis & DeNardo, 2007), as well as foraging behavior (i.e., dietary water intake, Lillywhite, 2017), and (c) *metabolic water production* (Chown, 2002; Stier et al., 2017). Some species are also capable of storing water and can thus use alternative sources of water (such as the bladder of desert species, Davis & DeNardo, 2009). Desiccation risks depend on water vapor pressure deficit and skin permeability, such that species differ tremendously in water loss rates through evaporation and therefore vulnerability to dehydration. For example, desiccation risk is a strong constraint on the water balance of amphibians that have a wet and permeable skin (Seebacher & Alford, 2002). Species also differ importantly in tolerance to dehydration, and many organisms can go for extent period of time in places without permanent access to water (e.g., in Gila monsters, Davis & DeNardo, 2007). Water constraints are not universal and are restricted to desiccating environments, such as terrestrial habitats and salt water, and thermoregulation is therefore virtually free from water limitation in freshwater or very humid environments. Desiccation risk depends on spatiotemporal patterns of air moisture, which is a nondepreciable resource that covary with environmental temperatures. Free‐standing water may also be limited in time and space in the environment (Owen‐Smith & Goodall, 2014), and this resource can be depleted especially when it is scarce and when the number of competitors increases, leading to enhanced exploitation as well as interference competition for water among individuals (Valeix, Fritz, Matsika, Matsvimbo, & Madzikanda, 2008).

Critical aspects of coupling between body temperature and water balance regulation have already been examined for endothermic vertebrates because these animals have high internal turn‐over rates for water and energy and may rely on evaporative cooling for heat dissipation at high ambient temperatures (see Figure 1). Studies of endotherms have focused on the consequences of this coupling for the thermal physiology of large mammals (Mitchell et al., 2018), the evolution of avian physiology and behavior in arid zones (Gerson, Smith, Smit, McKechnie, & Wolf, 2014; Tieleman & Williams, 2002a), the energetic balance of birds during migration (Klaassen, 2004), or patterns of daily torpor, hibernation and heterothermy in birds and mammals (Ruf & Geiser, 2015). On the other hand, it has been suggested that ectotherms must adjust their body temperature within a preferred range that depends on species' resistance to water loss and water availability in the environment (Angilletta, 2009). This is especially true for wet‐skinned ectotherms, such as amphibians, whose performances and physiology are strongly sensitive to dehydration (Anderson & Andrade, 2017). However, the thermal sensitivity and tolerance of ectotherms have received much greater attention so far in climate change ecology compared to physiological and behavioral responses to water balance regulation and tolerance to dehydration (Gunderson & Stillman, 2015; Huey et al., 2012; Woods, Dillon, & Pincebourde, 2015). In addition, both thermo‐ and hydroregulation processes have been typically studied independently in ectotherms, despite the potential interaction between them in driving ecological responses to climate change, which is highlighted in recent mechanistic models of their energy and water balance (Kearney & Porter, 2009; Pirtle, Tracy, & Kearney, 2019).

In this paper, we propose the unifying concept of thermo‐hydroregulation to emphasize this functional integration (i.e., the existence of statistical correlations and functional interactions among distinct phenotypic traits) between water balance and body temperature regulation. We define thermo‐hydroregulation as an interactive set of behavioral and physiological processes that maintain both water and thermal balance, and thereby “optimize” performances, survival, and reproduction. The concept of thermo‐hydroregulation encapsulates the idea that thermal conditions, humidity conditions, and water availability (as free‐standing water, dietary, or metabolic water) vary nonindependently (see above). In addition, because of interactions among the organismal processes most influential to water and thermal balance (Figure 1), thermoregulation and hydroregulation may involve shared behavioral (e.g., activity, exploration, habitat selection, or foraging) and physiological responses (e.g., cutaneous exchanges, maintenance costs, or resource storage) influenced by functional trade‐offs between the needs to concomitantly regulate water and heat balance. Focusing on terrestrial and semiterrestrial ectotherms, we briefly review the dominant thermo‐hydroregulation mechanisms in these taxa and identify some gaps in our current understanding of thermo‐hydroregulation. We next discuss the implications of thermo‐hydroregulation mechanisms for whole‐organism performances and ultimately fitness using an extension of the graphical cost–benefit model of thermoregulation in ectotherms (Angilletta, 2009; Huey & Slatkin, 1976). Eventually, we illustrate some important ecological and evolutionary consequences of thermo‐hydroregulation and propose guidelines for future studies.

## 2 FUNCTIONAL INTEGRATION OF THERMOREGULATION AND HYDROREGULATION IN ECTOTHERMS

Comparative physiologists have shown that thermoregulation and hydroregulation influence each other through several pathways with strong interspecific variation between the two dominant modes of thermoregulation (endothermy vs. ectothermy) but also within each thermoregulation mode (see Figure 1 and Boxes). Here, we focus on three different hierarchical levels of functional integration between thermoregulation and hydroregulation relevant to ectotherms in general: cutaneous and respiratory heat and water exchanges, behavioral regulation of thermal and water balance, and the coupling between the mass and water budgets of ectotherms.

### 2.1 Physiological thermo‐hydroregulation

Comparative studies have shown that thermal and water budgets are partly determined by biophysical properties of the body (e.g., area, coloration, shape, or surface‐specific resistance to water loss), which acts as an exchange surface for heat and water between the animal body and the environment. In terrestrial ectotherms, cutaneous evaporative water loss can be a significant contribution to total water loss and thermoregulation directly influences water balance because the water vapor pressure gradient between the animal and its environment increase with body temperature whereas surface‐specific resistance to water loss may decrease with body temperature in some species (Chown et al., 2011; Lourdais et al., 2017; Spotila, 1972). Ectotherms have evolved diverse indirect means to control rates of water loss such as mucus secretion in anurans (Lillywhite, 1971), changes in the lipid barrier to water in nonavian reptiles and amphibians (reviewed in Lillywhite, 2006), or changes in cuticular properties in insects (reviewed in Chown et al., 2011). On the other hand, water balance is variably influenced by respiratory water loss in terrestrial ectotherms. Ambient temperature generally increases body temperature, hence metabolism and breathing activity, which may lead to higher respiratory water losses because of the universal relationship between respiratory exchanges and transpiration (Woods & Smith, 2010). Overheating and dehydration risks can therefore select for functional adaptations such as a metabolic depression (Little & Seebacher, 2016; Muir, Costanzo, & Lee, 2007; Terblanche, Clusella‐Trullas, & Chown, 2010, see Table 1). The high diversity of respiratory modes in ectotherms makes it hard to generalize patterns of respiratory water loss responses to changes in thermal and water conditions (e.g., tracheal system vs. lungs; continuous vs. discontinuous respiration, see Chown, 2002, for examples in insects). Active mechanisms enabling the regulation of water balance and body temperature exist in endotherms such as panting in the face of environmental changes in temperature (Tieleman & Williams, 2002b) or the acute physiological regulation of evaporative water loss in the face of environmental changes in air humidity (Withers & Cooper, 2014). Those may also apply in some ectotherm species (Tattersall et al., 2006).

**Table 1:** Examples of classical, adaptive explanations of thermoregulation or hydroregulation strategies, and their re‐interpretation in the framework of thermo‐hydroregulation

	Examples	The classical explanation	The thermo‐hydroregulation perspective
Physiological regulation in ectotherms			
Metabolic depression	Downregulation of basal metabolism during acclimation to higher temperatures in lizards (Christian, Bedford, & Schultz, 1999)	*Thermoregulation*: Physiological and metabolic acclimation responses in order to save energy during thermoregulation	Metabolic depression is involved in both thermoregulation and the maintenance of an optimal hydration state through lower respiratory water loss
Critical thermal limits	Lethal body temperature limits of nonavian reptiles, amphibians, and insects (Angilletta, 2009)	*Thermoregulation*: These limits are the body temperature boundaries an individual should not pass when thermoregulating	CTLs would change if the organism is dehydrated. As heat would imply water losses, being dehydrated implies less resistance to heat
Metabolic water production	Production of water through catabolic pathways in flying insects (Chown & Nicolson, 2004)	*Hydroregulation*: Production of water to compensate water losses and dry food consumption	In high temperature environments, metabolic water production can overrides the water losses due to overheating and even participate in hives cooling in the case of eusocial hymenoptera
Behavioral regulation in ectotherms			
Activity patterns	Aestivation in desert lizards or reduced activity during hottest hours of the day (Porter, Mitchell, Beckman, & DeWitt, 1973)	*Thermoregulation*: Activity time dictated by availability of optimal operative temperatures in the environment with reduced activity when operative temperatures are above the optimum	A lower activity reduces the risks of overheating and dehydration by limiting exposure to warm and dry air conditions when free‐standing water is not available
Microhabitat selection	Use of thermal microhabitat to heat or cool down in insects (Caillon et al., 2014)	*Thermoregulation*: Microhabitat choice driven by spatial heterogeneity in operative temperatures and constraints on movement patterns to ideally select optimal body temperatures	Microhabitat selection explained by joint optimization of water loss, heat exchanges and energy expenditure, and nonenergetic factors. If water is limiting, optimal body temperatures for heat exchanges and energy metabolism may not be reached
Posture changes	Change in the posture according to the daytime in frogs (Pough et al., 1983)	*Hydroregulation*: Change in water availability selects for different body postures between day and night to reduce the rate of evaporative water loss	Posture changes though time are explained by the need of optimizing heat transfers and at the same time minimizing water losses

In ectotherms, basking activity (increased body exposure to solar irradiance through postural adjustment and microhabitat selection) is the dominant component of behavioral thermoregulation in heliothermic ectotherms (Angilletta, 2009), but it can also increase rates of evaporative water loss leading eventually to dehydration (Dupoué, Stahlschmidt, Michaud, & Lourdais, 2015; Lourdais et al., 2017). For example, Pirtle et al., 2019 reported that relevant shifts in basking time can increase the total water loss by up to 90% in scincid lizard species from Australia. As a consequence, water deprivation can cause thermal depression (i.e., behavioral preferences for lower body temperatures) to limit additional water loss as shown in some lizards, snakes, and amphibians (Anderson & Andrade, 2017; Ladyman & Bradshaw, 2003; Law & Bradley, 1990). We, however, expect strong differences among species with regard to this trade‐off between thermal preferences and water loss. In particular, there should be less opportunities for such a trade‐off in environments with plenty of thermal and water resources or, in the contrary, in environments with such limited resources that organisms have evolved to become either thermal specialists or water specialists. This is true for some insects' species with unusually strong resistance to evaporative water loss (Chown et al., 2011), strong tolerance to dehydration (Everatt, Convey, Bale, Worland, & Hayward, 2015; Kleynhans & Terblanche, 2011), or strong tolerance to hyperthermia (Everatt et al., 2015; Hoffmann, Chown, & Clusella‐Trullas, 2013; Hoffmann, Sørensen, & Loeschcke, 2003).

### 2.2 Behavioral thermo‐hydroregulation

Another set of mechanisms linking water budget and temperature regulation involves behavioral choices. On the one hand, in ectotherms, habitat humidity and individual water balance can influence daily and annual activity patterns, habitat selection or movement, leading to behavioral hydroregulation, whereby individuals flexibly adjust their behavior according to external and internal conditions to regulate their hydration state (Guillon et al., 2013). On the other hand, behavioral thermoregulation also involves changes in habitat choice decisions or activity patterns, and is the dominant mode of thermoregulation in ectotherms. It is thus anticipated that behavioral thermoregulation should interact with hydroregulation including behavioral tactics to reduce water loss (Kühnholz & Seeley, 1997; Pintor et al., 2016; Pirtle et al., 2019; Spotila, 1972) and water drinking behaviors (Davis & DeNardo, 2007). One possible outcome of this interaction is that a thermoregulatory strategy that “optimizes” performances does not minimize water loss or maximize water intake, which leads to a behavioral trade‐off between thermoregulation and hydroregulation (Davis & DeNardo, 2009; Grant, 1990). However, a positive feedback is also possible when behavioral selection of a body temperature that optimizes the energy budget also facilitates exploration and thus water finding in the landscape (Rozen‐Rechels et al., 2018), or when microhabitat selection favors jointly thermoregulation and water balance (e.g., shade‐seeking during hot days in reptiles; Pirtle et al., 2019). Unfortunately, most behavioral studies of thermoregulation have not considered effects of nonenergetic mechanisms related to water balance. Yet, recent evidence suggests that water availability in the environment can modify the costs and benefits of behavioral thermoregulation in ectotherms such as basking, foraging, or resting (Caillon et al., 2014; Woods et al., 2015). It is thus expected that thermal and water landscapes are critical determinants of behavioral patterns of thermo‐hydroregulation in spatially structured and fluctuating environments (Caillon et al., 2014; Sears et al., 2016; Woods et al., 2015).

The concept of thermo‐hydroregulation further questions the relevance of standard explanations of behavioral strategies in ectotherms (see Table 1). Basking decisions apparently related to the regulation of temperature and the energy budget could prioritize water conservation or be highly constrained by water conditions under some circumstances (e.g., seasonal activity depression during a summer drought, Davis & DeNardo, 2009). Non‐energetic costs of basking related to water balance and hydroregulation may also explain why many ectotherms prefer body temperatures lower than those that maximize energy intake and various measures of physiological performances related to the energetic status of the individual (Martin & Huey, 2008). Thus, basking behavior should be considered as a critical component of thermo‐hydroregulation and not solely thermoregulation in ectotherms.

### 2.3 Coupling the energy and water budgets

A third relevant set of mechanisms involve the tight coupling between the energy and water budgets (Kearney & Porter, 2009; Kearney, Simpson, Raubenheimer, & Kooijman, 2013). Body temperature directly influences the nutrient and energy budgets through temperature‐dependent physiological changes in ingestion rates, allocation rules, and process rates. The mass balance equation for water is mechanistically linked with those for nutrients and energy because food can provide both nutrients and water, energy catabolism produces water, and feces production combines both nutrient and water loss. Foraging behavior, prey selection, and metabolic water production are therefore important components of thermo‐hydroregulation. For example, “optimization” of resource acquisition is often considered as a critical target of thermoregulation behavior in ectotherms, but the same species may also rely on dietary water for maintaining water balance leading to dual effects of temperature and water balance regulation on foraging and prey selection (e.g., for insects: McCluney, 2017). Although metabolism is not a dominant input of water in most ectotherms in comparison with endotherms, some nondrinking insect species can increase their metabolic water production by consuming more stored energy during flight activities (Chown, 2002; Chown & Nicolson, 2004). These processes altogether determine the mass balance equations for nutrient–energy allocation into life‐history traits. Mechanistic models implemented to quantify simultaneously water and nutrient dynamics are therefore essential to predict the consequences of energy and water budgets on the life‐history traits and population dynamics of ectothermic species (Kearney, Munns, Moore, Malishev, & Bull, 2018; Kearney et al., 2013).

## 3 A CONCEPTUAL MODEL OF THERMO‐HYDROREGULATION

A fruitful avenue to understand variation in thermo‐hydroregulation in ectotherms is the cost–benefit approach proposed to model the adaptive evolution of thermoregulation by Huey and Slatkin (1976). The original model was designed to understand patterns of variation in thermoregulation primarily in lizards. It assumes that energy gains, whole‐organism performances and eventually fitness increase with body temperature until an optimum temperature is reached (see thermal performance curves in Figure 2a and Box 1) and that the benefits of thermoregulation are constrained by a range of energetic costs, defined as the energy expenditure needed to select the optimum body temperature for performance in a given environment. Generally, costs of thermoregulation increase as a function of the difference between environmental temperatures and body temperatures (Huey & Slatkin, 1976) and decrease with the heterogeneity of the thermal landscape (Sears & Angilletta, 2015; Sears et al., 2016). The cost–benefit model can further account for nonenergetic costs, such as predation risks (Dowd, King, & Denny, 2015), and has proven central in predicting patterns of behavioral thermoregulation and understanding the thermal biology evolution of ectotherms (Angilletta, Niewiarowski, & Navas, 2002).

We suggest that the cost–benefit model of thermoregulation can easily accommodate the functional integration of thermoregulation with hydroregulation. Irrespective of thermoregulation, hydroregulation results in fitness benefits associated with the homeostatic maintenance of an “optimal” water balance for whole‐organism performance, which are confined by the deleterious effects of extreme deviations in water balance (i.e., dehydration or overhydration leading to impairment of metabolism, muscular power, or brain capacities; Figure 2b). If the relationship between hydration state and performance was independent of body temperature, we would expect additive thermal and hydric performance curves and independent optimization of the functional traits related to temperature and water balance regulation (Figure 2c,d). However, fitness is likely better modeled by the interactive thermo‐hydroregulation process and the resulting three‐dimensional area of covariation in thermal and water performance curves (Figure 2e,f). Empirical support of such three‐dimensional benefit curves of thermo‐hydroregulation comes from experimental studies of locomotor performance curves of amphibians exposed to short‐term changes in body temperature and acute dehydration (Anderson & Andrade, 2017; Preest & Pough, 1989). In these species, modest to severe dehydration usually leads to a decrease in the maximal locomotor performance capacities as expected, but also a decrease in the optimal body temperature for performances as well as a reduction of the performance breadth and the thermal tolerance range. Thus, dehydration not only changes mean performance capacity but leads to a significant shift in the shape of thermal performance curves (Anderson & Andrade, 2017). One proximate explanation for nonadditive effects of water balance and body temperature on performance traits is that dehydration modifies the thermal sensitivity of cell and tissue metabolism as well as protection against thermal stress.

Not only can the benefits of thermoregulation and hydroregulation be nonadditive, but the costs of hydroregulation must also interact with the costs of thermoregulation in ectotherms. Hydroregulation costs should be primarily determined by spatiotemporal variations in environmental conditions most influential to water intake and loss rates such as wind speed, air moisture, and availability of free‐standing water. A higher effort and a more accurate thermoregulation also entail immediate energetic costs, such as when animals have to behaviorally select their habitats to maintain body temperature within the optimal range (Huey & Slatkin, 1976), and various nonenergetic costs, for example, when basking and habitat selection enhances predation risk (Angilletta, 2009; Box 1). When water resources are limiting, the bivariate cost–benefit model of thermo‐hydroregulation emphasizes the need to consider the additional costs of water balance regulation and interactions with thermoregulation. Whereas body temperature fluctuates quickly in ectotherms, especially in the smallest species, some of the costs of hydroregulation are likely delayed because the water balance changes more gradually as a function of water intakes and losses. The costs of hydroregulation are also likely more asymmetric than those of thermoregulation because they are essentially associated with avoidance of habitats with low water availability and high potential water loss rates (but see Chown & Nicolson, 2004 for situations where avoidance of overhydration is relevant in insects).

Behavioral ecologists often envision three different kinds of costs (opportunity, energy, and risks) of thermoregulation. Unfortunately, we still know extremely little about the costs of behavioral hydroregulation in ectotherms and can only speculate on their interactions with the costs of thermoregulation. Opportunity costs of thermo‐hydroregulation could imply a time trade‐off between investment in thermoregulation and hydroregulation. Increased basking effort allows ectotherms to reach faster their optimal temperature but can compromise their water balance on the long run, possibly reducing performance and thus growth and survival. For example, in lizards, the net effect of an increased behavioral activity in full sun on water balance is negative and not offset by potential positive effects of thermoregulation on metabolic water production and dietary water intake (Pirtle et al., 2019). Energy costs of thermo‐hydroregulation depend on the spatiotemporal distribution of operative temperatures, water sources (from food and free‐standing water), and potential water loss rates in the environment. When these are distributed nonindependently in the landscape, energy costs of thermo‐hydroregulation will therefore be lower or higher than the sum of thermoregulation and hydroregulation costs. Finally, when and where parasites, competitors, or predators are located in the environment will determine the risk costs of thermo‐hydroregulation. Measuring these three kinds of costs still remains a major challenge because evolutionary theory demonstrates that they depend on movement routines of animals, fine‐scale variation in environmental conditions, and broad‐scale distribution of resources and associated risks (Sears & Angilletta, 2015; Sears et al., 2016).

## 4 ECOLOGICAL AND EVOLUTIONARY IMPLICATIONS OF THERMO‐HYDROREGULATION IN ECTOTHERMS

As seen above, the need to thermo‐hydroregulate interacts with the distribution of microclimate and water resource within landscapes and determines how individuals use space, move, and disperse. Little is known on whether simultaneously or sequentially fulfilling both thermo‐ and hydroregulation needs can easily be achieved, or whether habitat selection trade‐offs exist because of the relative distribution of suitable habitats. The cost and benefits associated with resource prospecting have been better studied in endotherms, and we anticipate that movement and habitat choice patterns in ectotherms could follow similar rules (Bartelt, Klaver, & Porter, 2010; Sears et al., 2016). Studies of movements of ectotherms between water sources in arid or semiarid environments provide an excellent opportunity to address behavioral constraints induced by thermal and water availability, as seen with research done in ungulates (Cain et al., 2006). Areas near water sources could have more or less vegetation cover. This will affect the ability of individuals to find both basking spots, shade, and water easily. This suggests that not all habitats offer the same level of complementation with regard to thermo‐ and hydroregulation needs, where complementation is defined as the effect of the spatial distribution and accessibility of several limiting resources in the landscape on population abundance (Dunning, Danielson, & Pulliam, 1992).

Individuals do not necessarily have to endure environmental conditions in their local habitat, but they can also disperse to more suitable environmental conditions. Selection pressures due to the needs of thermo‐hydroregulation should favor the evolution of dispersal strategies to avoid both water and temperature stress. In the context of global climate change, the suitable thermal niches of many species are shifting toward the cold margin of their current distribution and dispersal becomes critical to allow organisms to track this shifting niche (Le Galliard, Massot, & Clobert, 2012). Global climate change could reduce complementation at the landscape scale when local warming is associated with higher drought frequencies and increase it when local warming is associated with higher rainfall, such as in temperate areas (Dore, 2005). Unfortunately, empirical studies of climate niche shifts and dispersal plasticity of ectotherms in response to joint needs for temperature and water are exceedingly rare. Experiments on short distance, natal movements in the common lizard (*Zootoca vivipara*), indicate that natal dispersal is enhanced in dry environments and in cold conditions, with additive but not interactive effects between the two factors (Massot, Clobert, & Ferrière, 2008; Massot, Clobert, Lorenzon, & Rossi, 2002). Thus, whether rising temperature will increase or reduce dispersal is not easy to predict in this species given a regional context of drier climate conditions in the coming years.

The concept of thermo‐hydroregulation has also major implications for life‐history evolution. To date, life‐history trade‐offs mediated by dual temperature and water needs are little investigated relative to energy‐based trade‐offs (Dupoué, Stahlschmidt, Michaud, & Lourdais, 2015; Lourdais et al., 2017). Temperature and water requirements are often elevated during reproduction and the thermo‐hydroregulation concept will help unravel trade‐offs shaping reproductive strategies. For example, parental care to the eggs and embryos has emerged repeatedly in invertebrates and vertebrates, and it is often stated that benefits of parental care are primarily derived from enhanced regulation of thermal conditions in ectotherms (Farmer, 2003; Shine, 2004). While we do not underestimate the benefits of parental thermoregulation to embryonic development, high temperature may also increase the rate of water loss from the eggs and maternal protection against desiccation appears widespread in insects (Ostwald, Smith, & Seeley, 2016; Smith, 1997), amphibians (Delia, Ramírez‐Bautista, & Summers, 2013), and nonavian reptiles (Poo & Bickford, 2013). For example, brooding behavior seems primarily related to minimizing egg water loss in pythons (Lourdais, Hoffman, & DeNardo, 2007), and the tight physical association between the mother and the clutch may have been a favorable context for the subsequent emergence of endothermy in these snakes (Shine, 2004). Therefore, the need of jointly regulating thermal and water balance during periods of high parental investment may influence the evolution of parental behaviors and brooding in ectotherms.

The thermo‐hydroregulation concept might also be useful in understanding parent–offspring conflicts traditionally envisioned in the framework of energy‐based allocation trade‐offs (Crespi & Semeniuk, 2004). Recent studies in viviparous lizards and snakes suggest that parent–offspring conflicts can be mediated by water demands of the progeny and can interact with parental thermal and water balance (Dupoué, Brischoux, et al., 2015; Dupoué et al., 2018). For example, female asp vipers maintain higher body temperature during pregnancy, which is beneficial to the embryos (Lorioux, Lisse, & Lourdais, 2013). Females also provide all the embryonic water and a foeto‐maternal conflict for water occurs if water resource becomes limiting, whereby females can alter their water balance to protect embryos from water stress (Dupoué et al., 2016). This situation may apply in other viviparous species of ectotherms as well. Parent–offspring conflicts are important evolutionary drivers of modes of reproduction and postnatal care, but most evolutionary hypotheses traditionally involve either thermal or energy constraints (Crespi & Semeniuk, 2004). We suggest that incorporating water requirements and interactions with parental thermoregulation is a crucial facet that has been neglected so far.

## 5 GUIDELINES FOR FUTURE STUDIES

**Figure 3:**
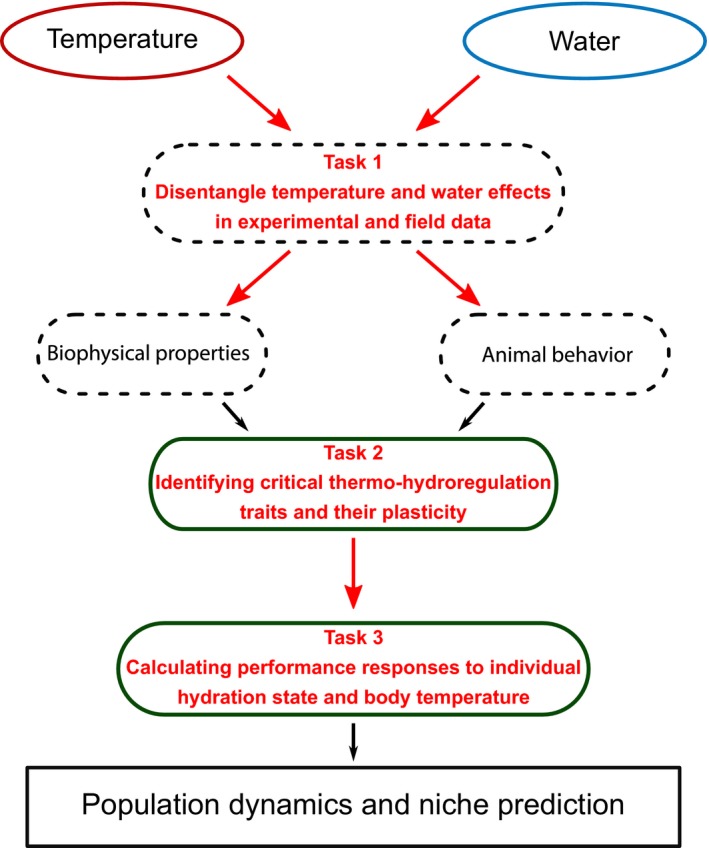
**Guidelines for future studies**. With this paper, we aim at proposing guidelines for future studies trying to understand the responses of ectotherms to changes in their temperature and water environment. We identified three levels of the integration of thermo‐hydroregulation processes that need critical experimental and empirical deepening. The first task would be to better disentangle the environmental temperature and water implications on microclimate properties. In the environment, a wet habitat is also often cooler than a dry one. Future studies should pay attention to be able to have all combinations of water and thermal environments or focus on other variables such as the biophysics of the microhabitat (evaporative water losses, operative temperature). A second task is to better understand the functional responses of ectotherms to these habitats. Physiological and behavioral responses to temperature are quite well‐known, but responses to water availability are still overlooked. We should now ask what mechanisms are common to thermoregulation and hydroregulation, and also investigate their plasticity and flexibility that could be critical in understanding organism responses to global changes. Finally, the last task aims at highlighting thermo‐hydroregulation performance curves in ectotherms taxa as they were only studied in anurans until now. Longer‐term performance studies are also needed to understand fitness consequences of environmental changes on ectotherms

We have shown above how thermoregulation and hydroregulation are functionally integrated in ectotherms and implications of the thermo‐hydroregulation concept for a range of ecological and evolutionary processes due to potential trade‐offs between the needs to regulate heat and water balance concomitantly. The cost–benefit model of thermo‐hydroregulation suggests that an important avenue for future research should try to link environmental variation with concomitant changes in individual hydration state and body temperature as well as consequences for individual performances and costs of thermo‐hydroregulation. Here, we propose a step‐by‐step methodology to improve our understanding of thermo‐hydroregulation strategies in ectotherms (see Figure 3).

The thermo‐hydroregulation concept raises the need for studies that better account for environmental conditions relevant to both water balance and body temperature regulation (step 1 in Figure 3). Eventually, such studies should try to disentangle the physiological and ecological effects of thermal and hydric conditions including additive and interactive effects of multiple environmental factors. In particular, we need more investigations of the influence of environmental temperatures on water balance and of the influence of hydric conditions on the thermal biology. To disentangle the effects of temperature and water in natural habitats, it will also be important to move beyond current research practices, where species or populations are usually compared across “hot and dry” environmental gradients without knowing which environmental factor drive adaptations of thermo‐hydroregulation strategies (Cox & Cox, 2015; Huang, Talal, Ayali, & Gefen, 2015). At least three different solutions are available. First, empirical studies should include detailed descriptions of both thermal and hydric conditions, including potential evaporative water loss rates and water availability in the environment. For example, biophysical models currently make it possible to produce maps of operative temperature and evaporative water loss for ectotherms across a wide range of spatial scales (Bartelt et al., 2010). These maps, together with conceptual frameworks such as the colimitation theory (Koussoroplis, Pincebourde, & Wacker, 2017), could then be used to predict functional traits and performances of ectotherms in fluctuating environments. Second, comparisons across environmental gradients in target species should focus on independent geographic variation in water and thermal conditions to reduce colinearity between these environmental factors. This is likely to be feasible at a regional scale by combining climate gradients (e.g., across a latitudinal or altitudinal clines) with local variation in water availability in the habitat (Dupoué et al., 2017). Third, one can also rely on classical factorial experimental design in the field (e.g., temperature cline combined with a rainfall manipulation, Kreyling et al., 2018) or in the laboratory with climate chambers. Controlled environment facilities indeed allow the detailed and independent control of temperature, water vapor density, and water availability and make it possible to quantify the physiological and behavioral sensitivity of ectotherms to heterogeneous environments (Riddell, McPhail, Damm, & Sears, 2018).

The thermo‐hydroregulation concept also praises for more integrative functional analyses of ectotherms' sensitivity to changes in their environment (step 2 in Figure 3). To elucidate the joint physiological and behavioral mechanisms of body temperature and water balance in ectotherms, we need a stronger emphasis on hydroregulation strategies than in current climate change research (Sinclair et al., 2016, but see Kearney et al., 2018) and a better integration of functional studies of hydroregulation and thermoregulation. Future investigations should aim at identifying the most critical functional traits involved in thermo‐hydroregulation and how these traits can allow acclimation and adaptation of ectotherms to their changing environment. Biophysical models and analyses can be used to rank physiological and behavioral traits according to their relevance for body temperature and water balance regulation, and to quantify if these traits are involved in functional trade‐offs between, for example, water loss and thermoregulation. For example, such models applied to terrestrial lizards predict that changes in the skin properties and behavioral tactics are a much more important contribution for hydroregulation in response to changes in water availability than metabolic changes (Pirtle et al., 2019). In addition, bioenergetic models require a good understanding of all water balance mechanisms including dietary and metabolic water inputs. We thus need to improve our basic knowledge of foraging behavior and the relationship between water balance and diet as well as catabolism (Brusch, Lourdais, Kaminsky, & DeNardo, 2018; Chown & Nicolson, 2004; Wright, Jackson, & DeNardo, 2013). Analogous to studies on endotherms, we also need empirical tests of the acclimation and adaptation responses of thermo‐hydroregulation traits in ectotherms (Cain et al., 2006). Multivariate analysis of acclimation responses should focus on metabolic depression, thermal depression, and cutaneous resistance to water losses since those traits are likely critical in the thermo‐hydroregulation strategies of ectotherms (Chown et al., 2011; Little & Seebacher, 2016).

Another important avenue of research would be to better characterize thermo‐hydroregulation behaviors and their plasticity. There is indeed great scope to improve our understanding of the behavioral responses of ectotherms to variation in water availability and hydric conditions relative to thermal conditions, and to disentangle temperature and water effects on behavior (Kearney et al., 2018; Pintor et al., 2016; Rozen‐Rechels et al., 2018). This is particularly pressing because habitat selection mechanisms are of great importance to both temperature and water balance regulation (Pintor et al., 2016) but still remain a black box in predictive models of ectotherms' populations (Kearney & Porter, 2009; Kearney et al., 2009). Laboratory experiments with shuttle boxes or contrasted microhabitats (Pintor et al., 2016) and field studies of individual movements in habitat landscapes (Bartelt et al., 2010; Sears et al., 2016) will be crucial to make significant progress in this direction and to quantify the potential of behavioral traits to buffer environmental change effects. The ecological consequences of individual thermo‐hydroregulation mechanisms could depend on the social context because the link between performances and population dynamics is not completely straight forward (Figure 1). In order to quantify the importance of social competition and facilitation study of thermo‐hydroregulation, mechanisms at the population level are needed. Promising avenue in this perspective would be to use new generation mesocosms set‐ups allowing partial control of environmental factors (e.g., rainfall, temperature) in seminatural conditions (Legrand et al., 2012; Sears et al., 2016).

Finally, the thermo‐hydroregulation concept calls for more systematic empirical tests of the concurrent effects of hydration state and body temperature on individual performances, fitness, and eventually ecological processes such as population growth (step 3 in Figure 3). Unfortunately, empirical examples of the three‐dimensional benefit curves of thermo‐hydroregulation are scant in ectotherms except for those obtained from acute stress experiments with anurans (see previous section and Figure 2, Anderson & Andrade, 2017; Preest & Pough, 1989). One reason is the low number of tests of the effects of water stress and hydration state on whole‐organism performance and lifetime fitness compared to effects of thermal conditions (Angilletta, 2009, but see, e.g., McCluney & Date, 2008). An explanation for this is that direct manipulations and quantitative measures of hydration state are more difficult to perform than those of thermal conditions and body temperature in ectotherms, in which thousands of thermal performance curves have been quantified (Angilletta, 2009; Angilletta et al., 2002). Controlled protocols to manipulate water balance, for example, changes in water availability, air moisture, or diet (see Dupoué et al., 2018; Dupoué, Brischoux, et al., 2015), should be designed, tested, and used to quantify hydric performance curves. Current examples of thermo‐hydroregulation in ectotherms only integrate short‐term, immediate interactions between body temperature and water balance (see Anderson & Andrade, 2017 or Pintor et al., 2016). We thus need to prioritize research that examines performances in the long‐term with controlled conditions, for example, using climate chambers, or in more complex, variable natural settings, for example, with coupled measurements of body temperature and hydration state in wild animals.

## 6 CONCLUSIONS

The physiological and behavioral regulation of body temperature and water balance should be considered as an integrated functional property of terrestrial and semiterrestrial ectotherms. Future studies should therefore focus on improving our understanding of the proximate mechanisms of joint water balance and body temperature regulation in contrasted environments and species, which may help to unravel the functional traits most likely to reflect variation in thermoregulation strategies and the most relevant trade‐offs between temperature and water balance regulation. We also need a better understanding of the benefits and costs of thermo‐hydroregulation including studies of thermal and hydric performance curves, detailed analyses of behavioral budgets, and lifetime fitness measurements in contrasted environments for water balance and temperature regulation. Future studies of habitat choice, dispersal strategies, and life‐history traits in ectotherms will benefit from a detailed knowledge of thermo‐hydroregulation and development of mechanistic models, which will help improve the predictions of ecological responses to future climate conditions.

## CONFLICT OF INTEREST

None declared.

## AUTHOR CONTRIBUTION

J‐FLG, DR‐R, OL, and AD conceived the idea. The content and structure of the paper was conceived collectively by all authors. DR‐R, J‐FLG, and AD led the writing of the manuscript. OL, SC‐J, and JC wrote some parts of the manuscript. DR‐R developed the figures, based on ideas from OL, SM, J‐FLG and contributions from all authors.
